# Ataluren-mediated nonsense variant readthrough in D-bifunctional protein deficiency: A case report

**DOI:** 10.1016/j.ymgmr.2024.101137

**Published:** 2024-08-29

**Authors:** Rai-Hseng Hsu, Ni-Chung Lee, Hui-An Chen, Wuh-Liang Hwu, Wang-Tso Lee, Yin-Hsiu Chien

**Affiliations:** a Department of Pediatrics, National Taiwan University Hospital, Taipei, Taiwan; b Department of Medical Genetics, National Taiwan University Hospital, Taipei, Taiwan; c Department of Pediatrics, National Taiwan University College of Medicine, Taipei, Taiwan; d Center for Precision Medicine, China Medical University Hospital, Taichung, Taiwan

**Keywords:** D-bifunctional protein deficiency, Readthrough therapy, PTC124 (ataluren), Uniparental disomy

## Abstract

D-bifunctional protein (DBP) deficiency, a fatal peroxisomal enzyme disorder, typically manifests with life-threatening symptoms in the first two years of childhood. We present the case of an infant with elevated lysophosphatidylcholine C26:0 (C26:0-LPC) levels identified during X-linked adrenoleukodystrophy (ALD) screening, leading to a diagnosis of DBP deficiency due to a homozygous *HSD17B4* c.1041T>A, p.(Tyr347Ter) variant. Starting at two months of age, the infant experienced seizures, hypotonia, and developmental delays, prompting the initiation of experimental treatment with the readthrough agent PTC124 (ataluren) at six months. The treatment led to a decrease in C26:0-LPC levels from 0.65 μM to 0.53 μM; concomitant fish oil supplementation transiently increased C26:0-LPC to 0.74 μM before returning to 0.53 μM after cessation of supplementation. The patient demonstrated improved swallowing and progressive motor and speech development during a two-year treatment period, with no further seizures. This case report highlights the potential of nonsense readthrough therapy for peroxisomal disorders, a group of metabolic diseases that currently lack targeted treatments.

## 1 Introduction

D-bifunctional protein (DBP) deficiency is a fatal autosomal recessive peroxisomal enzyme deficiency disease characterized by hypotonia, seizures, craniofacial dysmorphisms, psychomotor delay, deafness, and blindness in the neonatal period, with mortality generally occurring by two years of age [1]. DBP deficiency is caused by biallelic variants of the *HSD17B4* gene and currently has no effective treatment. In Taiwan, the Newborn Screening Center at National Taiwan University Hospital implements screening protocols for X-linked adrenoleukodystrophy (X-ALD) [2], which entail measuring lysophosphatidylcholine C26:0 (C26:0-LPC) levels. Elevated C26:0-LPC levels indicate several other peroxisomal diseases, including peroxisomal biogenesis disorders such as Zellweger spectrum disorder and deficiencies in peroxisomal enzymes or transporters, including DBP deficiency [3,4]. In this report, we describe the case of a male infant diagnosed with DBP deficiency through newborn screening and the follow-up and subsequent treatment by readthrough therapy.

## 2 Case report

A male infant was born to nonconsanguineous parents after 38 weeks of uneventful gestation, with a birth weight of 3190 g. Prenatal karyotyping of amniotic fluid cells, performed due to advanced maternal age, was normal. However, newborn screening revealed elevated levels of C26:0-LPC (0.775 μM, as determined by second-tier high-performance liquid chromatography analysis, normal <0.4 μM). Exome sequencing (ES) identified a novel homozygous *HSD17B4* c.1041T>A, p.(Tyr347Ter) variant. The father was heterozygous for the c.1041T>A variant, and it was not present in the mother. Further analysis using AutoMap [5], a homozygosity mapping tool, revealed a paternal uniparental disomy (UPD) of chromosome 5 spanning 216.28 Mb, which included the *HSD17B4* and *SLC45A2* genes. The infant also had a homozygous *SLC45A2* c.147C>G, p.(Tyr49Ter) variant, which caused oculocutaneous albinism type 4 (OCA4). OCA4 is characterized by hypopigmentation of hair, skin and eyes, as well as other ophthalmological abnormalities, including nystagmus, reduced visual acuity, and misrouting of visual pathways [6]. The *HSD17B4* variant accounted for his light hair and skin color, although no significant ocular or visual changes were identified.

Shortly after birth, the infant exhibited facial dysmorphisms, including frontal bossing, hypertelorism, depressed nasal bridge, long philtrum, and large ears, as well as light hair and skin pigmentation. At one and two months of age, he experienced focal seizures, and an electroencephalogram (EEG) revealed bilateral posterior temporal and right temporal discharges with excessive slow waves. Magnetic resonance imaging of the brain was unremarkable.

**Fig. 1:**
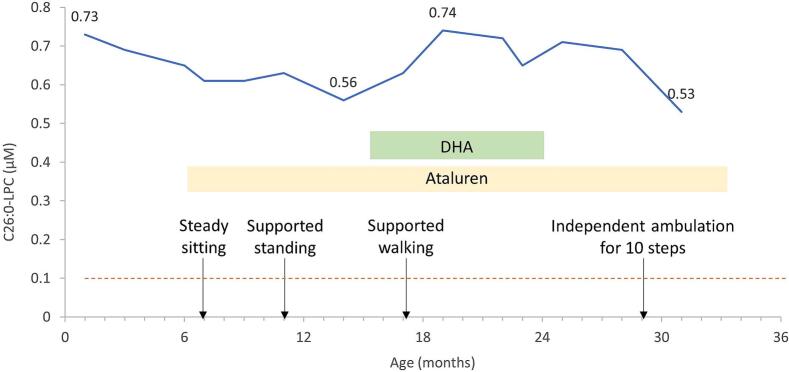
Trends in C26:0-LPC level, motor development, and treatment duration.

Owing to hypotonia, a propensity for easy choking, and developmental delays, experimental treatment with PTC124 (ataluren) was initiated at 6 months of age. This treatment led to a reduction in C26:0-LPC levels, which gradually decreased from 0.65 μM to 0.56 μM (Fig. 1). At 15 months of age, the parents began administering a fish oil supplement containing docosahexaenoic acid (DHA) and phosphatidylserine (Lovita), varying from 105 mg to 225 mg daily, due to concerns about DHA deficiency and its association with developmental delay [7,8]. This supplementation coincided with an increase in C26:0-LPC levels to 0.74 μM. After discontinuing the fish oil, the C26:0-LPC level decreased to 0.53 μM at 31 months.

This figure depicts the declining trend in C26:0-LPC levels during ataluren treatment alongside the continuous improvement in motor development. The normal cutoff value for neonates is <0.4 μM, and for infants, children and adults, it is <0.1 μM (indicated by the dashed line).

Following ataluren therapy, the patient's swallowing function improved. His motor skills progressed to independently sitting at eight months, supported standing at eleven months, supported walking at seventeen months, and unassisted walking for ten steps at 31 months. The patient experienced no further seizures, and an EEG examination at 12 months was normal. The Peabody Developmental Motor Scales – Second Edition evaluation at 25 months indicated normal fine motor development (quotient of 85, 16th percentile), but his gross motor development remained delayed (quotient of 64, <1st percentile). Although language expression was delayed, comprehension was within normal ranges, and auditory function was preserved. The patient also exhibited photophobia due to albinism.

## 3 Discussion

This case illustrates the utility of X-ALD screening, including the measurement of DBS C26:0-LPC levels, which provides an additional opportunity to detect and manage DBP deficiency early. The use of ES upon the discovery of elevated C26:0-LPC levels allowed for a definitive diagnosis despite the unusual molecular mechanism of this patient's *HSD17B4* variants, which involved paternal UPD. After failing to detect the variant in the mother, the patient's homozygous status for *HSD17B4* was elucidated using AutoMap, a high-performance homozygosity mapping tool. The concomitant diagnosis of *SLC45A2-*associated albinism due to UPD was pertinent to genetic counseling for the family. While X-ALD is the main targeted peroxisomal disease identifiable through C26:0-LPC screening, this case presents a possibility for therapeutic intervention in other peroxisomal diseases with genetic variants that involve nonsense changes.

Patients with DBP deficiency type I or the neonatal-onset form have hypotonia onset in the first month, barely acquire any psychomotor development, and typically do not survive beyond 14 months of age [9,10]. Most of these cases involve deletions, insertions, or nonsense variants [9]. The DBP spectrum now extends to type 2 and type 3, and most patients with these types have normal *very long chain fatty acids (*VLCFA) levels [11]. Our patient, with a homozygous nonsense variant and seizure onset at the age of 2 months, is predicted to be a type I patient. In addition, owing to the potential correlation between several biochemical parameters and the survival of DBP patients, we chose C26:0-LPC as the disease treatment biomarker since its overproduction has been positively associated with disease development [12].

Ataluren, which facilitates ribosomal readthrough of mRNAs containing premature stop codons, enables the synthesis of full-length proteins. This drug is approved for Duchenne muscular dystrophy [13,14] and is under investigation for other genetic disorders, such as cystic fibrosis [15], mucopolysaccharidosis type 6 [16], and mucopolysaccharidosis type 1 [17]. In patient-derived cellular models of PEX-related peroxisomal disorders [18], G418 (geneticin), another therapy based on nonsense suppression, improved VLCFA catabolism. In our case, our patient presented with a homozygous nonsense variant, seizures, and facial dysmorphisms [1], which are typical of the classic form of type I peroxisomal biogenesis disorders [10]. While this disorder is associated with seizures and neurodegeneration [1] and generally has a poor prognosis, our patient experienced no further seizures after ataluren treatment and exhibited progressive motor and cognitive development, in addition to stabilized C26:0-LPC levels. Although fish oil supplementation temporarily complicated the interpretation of C26:0-LPC levels, it did not appear to have harmed the patient since no developmental stagnation or regression was noted. Long-term monitoring is warranted due to the potential interference of nutritional supplements with C26:0-LPC levels.

Moreover, despite a nonsense variant in the *SLC45A2* gene, there was no discernible improvement in the patient's pigmentation following treatment with ataluren. Clinical experiences suggest that although preclinical studies on readthrough agents have been promising, the outcomes in clinical trials can be inconsistent [19]. This discrepancy is often attributed to the influence of the specific stop codon context on readthrough efficacy [20]. The observed variability in responses supports the theory that each nonsense variant needs to be tested by readthrough therapies individually.

## 4 Conclusion

This report underscores the potential of readthrough therapy in individuals with peroxisomal disorders caused by nonsense variants for which established treatments are lacking. Although readthrough strategies may have only limited utility for DBP deficiency patients, they still have promise as potential management options, as demonstrated in our case. This case also emphasizes the importance of early diagnosis, which is now more feasible in peroxisomal disorders due to newborn screening or other screening strategies, in the management and therapeutic intervention of peroxisomal disorders.

## Funding

None.

## Ethical compliance

The study was performed in accordance with the ethical standards of the Institutional Review Board of National Taiwan University Hospital. The need for written informed consent was waived for this chart review study.

## CRediT authorship contribution statement

**Rai-Hseng Hsu:** Writing – original draft, Formal analysis, Data curation. **Ni-Chung Lee:** Data curation, Conceptualization. **Hui-An Chen:** Conceptualization. **Wuh-Liang Hwu:** Conceptualization. **Wang-Tso Lee:** Conceptualization. **Yin-Hsiu Chien:** Writing – review & editing, Supervision, Data curation, Conceptualization.

## Declaration of competing interest

The authors declare that they have no conflicts of interest.

## Data Availability

Data will be made available on request.
